# Survival Wisdom of *Tradescantia spathacea* Sw: Trade-Off Between Anthocyanin-Mediated Photoprotection and Photosynthetic Capacity Under High-Light Stress

**DOI:** 10.3390/ijms27146492

**Published:** 2026-07-22

**Authors:** Xiaoting Zheng, Qingyi Cai, Hui Zhu, Yuzhong Zheng, Ziyuan Li, Kaicheng Luo, Peikun Xu, Zhengchao Yu

**Affiliations:** School of Life Sciences and Food Engineering, Hanshan Normal University, Chaozhou 521041, China

**Keywords:** *Tradescantia spathacea*, anthocyanins, photoprotection, photosynthetic capacity, antioxidant substances, transcriptome and light stress

## Abstract

Light heterogeneity shapes plant survival, yet how shade-tolerant species balance photoprotection and photosynthesis under changing light remains unclear. *Tradescantia spathacea* Sw, with green adaxial and purple-red abaxial leaf surfaces, exhibits both shade tolerance and high-light acclimation, but the functional specialization of its leaf sides and the underlying trade-off mechanisms are poorly understood. This study exposed *T. spathacea* to 100% (FL) and 30% (LL) sunlight for 2 years and systematically assessed light adaptation mechanisms through morphological observation, physiological and biochemical detection, and transcriptomic analyses. Results showed that under FL conditions, leaf morphology underwent adaptive remodeling. Anthocyanins accumulated specifically on the abaxial side, thermal dissipation and antioxidant systems were fully activated, while photosynthetic capacity was markedly inhibited, reflecting a survival-priority strategy. Under LL conditions, leaves became broader and flatter, chlorophyll content increased, and the expression of photosynthesis-related genes was upregulated. This enhanced light capture and carbon assimilation efficiency, leading to significantly greater biomass accumulation, which reflected a growth-oriented resource allocation strategy. Transcriptomic and physiological data consistently showed that FL upregulated anthocyanin biosynthetic genes, while LL activated photosystem and photosynthetic electron transport genes. *T. spathacea* achieves a flexible photoadaptation strategy across different light environments through leaf surface functional specialization and an anthocyanin-mediated trade-off between photoprotection and photosynthesis.

## 1. Introduction

Light is a core environmental factor regulating plant photosynthesis, growth, development, and metabolic processes [1], and provides the energy required for plant dry matter accumulation [2]. The effect of light intensity on plants exhibits a clear duality: optimal light levels enhance photosynthetic efficiency and normal growth, whereas excessive light leads to energy oversaturation, inducing photoinhibition and oxidative stress, and in severe cases, causes irreversible damage to the photosynthetic apparatus [3,4]. Under high-light conditions, the excitation energy absorbed by chloroplasts cannot be fully utilized by photochemical reactions, resulting in over-reduction in the photosynthetic electron transport chain and consequent overproduction of reactive oxygen species (ROS), including superoxide anion, hydrogen peroxide, and singlet oxygen [5]. These ROS persistently damage the D1 protein of photosystem II (PSII), thylakoid membrane lipids, and photosynthetic pigments, leading to PSII dysfunction, reduced photochemical efficiency, and membrane lipid peroxidation [6]. Long-term high-light stress disrupts the dynamic balance between D1 protein synthesis and degradation, creating a vicious cycle of photoinhibition that ultimately results in decreased net photosynthetic rate (Pn), leaf damage, and even plant mortality [6,7]. Tolerance to high-light stress varies significantly among plant species. Shade-tolerant plants generally exhibit thinner leaves, lower light saturation points, and reduced chlorophyll content, rendering their photosynthetic apparatus far less tolerant to high light than that of sun-tolerant plants [8]. When shade-tolerant plants are exposed to full sunlight, they are highly susceptible to PSII inactivation, ROS bursts, and exacerbated membrane lipid peroxidation [9]. Consequently, high-light stress represents a major threat to the survival and population regeneration of shade-tolerant plants.

To mitigate photodamage under high light and maintain the stability of the photosynthetic apparatus, plants have evolved a multi-layered, cooperative photoprotective system during long-term evolution. This system establishes a complete defense pathway that encompasses light filtration, excess energy dissipation, and reactive oxygen species (ROS) scavenging [10,11]. Plants can reduce light input to mesophyll cells by adjusting leaf angles, relocating chloroplasts within cells [10], and accumulating light-absorbing compounds in the epidermis [3]. Under high light, altering leaf orientation effectively lowers the photon flux density per unit leaf area, while chloroplast migration to the anticlinal cell walls minimizes light capture [10]. Furthermore, the accumulation of anthocyanins in the epidermis acts as a natural light screen, absorbing blue-violet and ultraviolet light, thereby effectively alleviating the light stress on chloroplasts [12]. This mechanism is particularly crucial for shade-tolerant plants with simpler anatomical structures. When light capture regulation alone is insufficient to dissipate excess light energy, plants activate the xanthophyll cycle-mediated non-photochemical quenching (NPQ) to safely dissipate the surplus excitation energy as heat [13,14]. Due to its rapid response and high reversibility, NPQ serves as a core strategy for plants to cope with short-term high-light stress [15]. However, even after these regulatory mechanisms are engaged, a residual amount of ROS may still be generated within cells. At this stage, the enzymatic and non-enzymatic antioxidant defense systems act in concert to quench these ROS [4,16]. Enzymatic components, such as superoxide dismutase, peroxidase, and catalase, directly scavenge ROS [4]. Meanwhile, non-enzymatic small-molecule antioxidants, including flavonoids, total phenolics, ascorbate, and glutathione, help maintain cellular redox homeostasis by directly quenching free radicals or serving as cofactors in antioxidant enzyme reactions [17,18].

In addition to the immediate physiological defenses mentioned above, plants also acclimate to different light environments through long-term morphological, developmental, and growth strategy remodeling [19]. Such acclimation, underpinned by the regulation of gene expression, reallocation of metabolic resources, and modification of plant architecture, has far greater ecological consequences than short-term physiological responses and plays a pivotal role in determining plant niche occupancy and biogeographical distribution [20,21]. Under low-light conditions, plants increase leaf area and specific leaf area, reduce leaf thickness, and adjust leaf angles to a more horizontal position, thereby maximizing the capture of limited light energy [22,23,24]. Concurrently, they upregulate the expression of genes involved in chlorophyll biosynthesis [25], photosystems I and II, and the photosynthetic electron transport chain [21], leading to increased chlorophyll content and an abundance of light-harvesting proteins [26,27]. These adjustments enhance light absorption and conversion efficiency, ensuring normal carbon metabolism and supporting plant growth and reproduction under low light [25]. By contrast, in high-light habitats, plants develop thicker leaves with more developed palisade tissue [28], which not only prolongs the light path within the leaf but also provides additional space for the accumulation of photoprotective compounds. At the same time, they increase leaf angles to a more erect position, reducing the amount of light intercepted per unit leaf area and alleviating photosystem II (PSII) stress [22]. Physiologically, plants enhance both thermal dissipation and antioxidant capacity [6]. At the molecular level, they upregulate key genes involved in anthocyanin biosynthesis, promoting the accumulation of protective pigments [29,30], while downregulating the expression of photosynthesis-related genes, thereby redirecting metabolic resources toward stress defense [31]. Although this strategy partially suppresses growth rate, it ensures the long-term survival of plants in high-light environments.

*T. spathacea,* a member of the Commelinaceae family, is a perennial evergreen herbaceous plant characterized by a short, condensed stem and clustered leaves. This species thrives in warm, humid, and semi-shaded environments and is naturally distributed along forest edges, stream banks, and shaded thickets. It exhibits excellent shade-tolerance while also being capable of adapting to full sunlight exposure. *T. spathacea* grows well in loose, fertile, well-drained sandy loam soils, with a pH adaptation range of 5.5–7.5. It has been widely used in landscaping as a high-quality shade-tolerant foliage plant. *T. spathacea* possesses typical dorsiventrally heterochromatic leaves (with green adaxial and purple-red abaxial leaf surfaces) and exhibits both shade-tolerance and adaptability to high-light environments, making it an ideal model for investigating plant photoadaptation mechanisms. However, the functional differentiation between the adaxial and abaxial side, as well as the intrinsic mechanisms underlying the trade-off between survival and growth under high- and low-light conditions, remains poorly understood. To address these gaps, this study established two light treatments: full sunlight (100% natural light) and 30% of full sunlight. Using a comprehensive approach integrating morphological observations, physiological and biochemical assays, chlorophyll fluorescence analysis, gas exchange measurements, and transcriptome sequencing, we focused on three key scientific questions: (1) What morphological and physiological strategies does *T. spathacea* adopt under different light intensities? (2) How does light intensity regulate the accumulation of abaxial side anthocyanins, and what is their photoprotective function? (3) Does the dorsiventral structural specialization enable spatial decoupling and trade-off between photoprotection and photosynthetic function? This study aims to elucidate the adaptive mechanisms of *T. spathacea* to heterogeneous light environments, contribute to the theoretical framework of shade-tolerant plant photoadaptation, and provide practical references for light management in ornamental horticulture.

## 2. Results

### 2.1. Growth Phenotype, Single Plant Fresh Weight, and Anthocyanin Content in Leaves of T. spathacea Under FL and LL Environments

**Figure 1:**
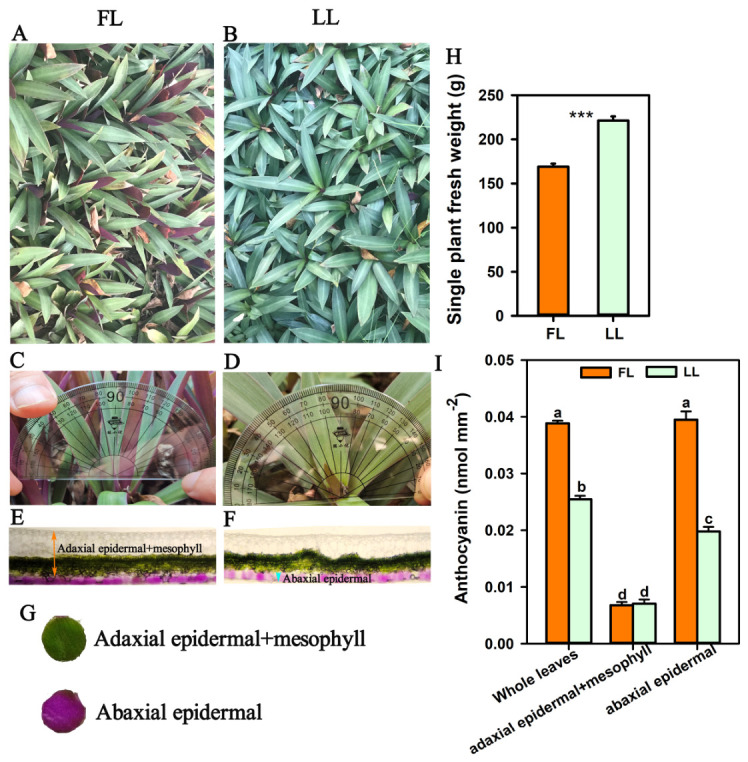
Growth phenotypes, angles between newly matured leaves and the ground, leaf cross-sections, adaxial and abaxial side phenotypes, single plant fresh weight, and leaf anthocyanin content of *T. spathacea* under FL and LL conditions. (**A**,**B**) Overall phenotypic comparison of *T. spathacea* under FL and LL treatments: left, plants under FL; right, plants under LL. (**C**,**D**) Measurement of the angle between newly matured leaves and the ground using a protractor (*n* = 19). (**E**,**F**) Microscopic observation of leaf cross-sections. (**G**) Phenotypes of the adaxial epidermal layer + mesophyll and the abaxial epidermal layer of leaves. (**H**) Fresh weight per plant of *T. spathacea* under FL and LL conditions (*n* = 11). (**I**) Anthocyanin content in different parts or whole leaves (*n* = 5). All data are presented as mean ± standard error (SE). *** indicates significant difference at the 0.001 level. Different letters above bars indicate statistical significance (*p* < 0.05).

Whether grown under FL or LL conditions, the leaves of *T. spathacea* exhibit a green adaxial and a red abaxial side (Figure 1A–G). Additionally, compared to those grown under FL, plants grown under LL conditions displayed greener leaves with greater leaf length and width (Figure 1B), as well as significantly higher individual plant biomass (Figure 1H). Using a protractor for precise measurement and observation, it was found that newly matured leaves of plants grown under FL had a larger angle relative to the ground and were more erect (Figure 1C,D). Furthermore, when comparing leaf anthocyanin content between FL and LL conditions, leaves under FL exhibited significantly higher anthocyanin levels than those under LL (Figure 1I). Notably, by mechanically peeling leaf samples into two parts, one consisting of the adaxial epidermal layer + mesophyll cells, and the other consisting of the abaxial epidermal layer (Figure 1G), and subsequently measuring anthocyanin content in each part, the results showed that no anthocyanin accumulation was detected in the adaxial epidermal layer + mesophyll fraction under either FL or LL conditions. In contrast, anthocyanins accumulated exclusively in the abaxial epidermal layer, with significantly higher accumulation under FL than under LL (Figure 1I). Microscopic observation of leaf cross-sections further confirmed that the adaxial side of the leaf is green and the abaxial side is red, with greater anthocyanin accumulation observed under FL conditions (Figure 1E,F).

### 2.2. Morphological Structure and Photosynthetic Physiological Characteristics of T. spathacea Leaves in FL and LL Environments

**Figure 2:**
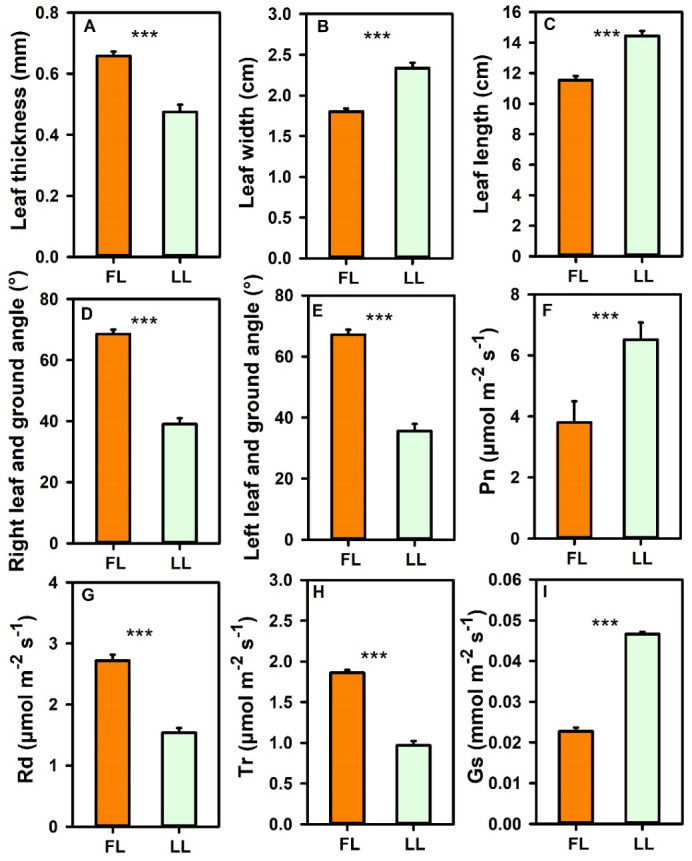
Changes in leaf morphological structure and photosynthetic physiological characteristics of *T. spathacea* under FL and LL conditions. (**A**–**C**) Leaf thickness, leaf width, and leaf length under FL and LL conditions, respectively (*n* = 11). (**D**,**E**) Angles of the left and right leaves relative to the ground (n = 19). (**F**–**I**) Net photosynthetic rate (Pn), respiration rate (Rd), transpiration rate (Tr), and stomatal conductance (Gs), respectively (*n* = 5). *** indicates significant difference at the 0.001 level.

To investigate the effects of different light intensities on leaf morphology and photosynthetic physiological characteristics of *T. spathacea*, this study conducted quantitative analyses on plants under FL and LL conditions. The results showed that, in terms of leaf morphology, leaf thickness was significantly greater in the FL group than in the LL group, whereas leaf width and leaf length were significantly greater in the LL group than in the FL group (Figure 2A–C). Notably, the angles between the leaves and the ground were significantly larger in the FL group, approaching approximately 80°, while leaves in the LL group were considerably flatter, with angles of around 40° relative to the ground (Figure 2D,E). Regarding photosynthetic physiological characteristics, the Pn and Gs of the FL group were significantly lower than those of the LL group. Conversely, the dark respiration rate (Rd) and transpiration rate (Tr) were significantly higher in the FL group than in the LL group (Figure 2F–I).

### 2.3. Chlorophyll Fluorescence Parameters and Antioxidant Substances in Leaves of T. spathacea Under FL and LL Environments

**Figure 3:**
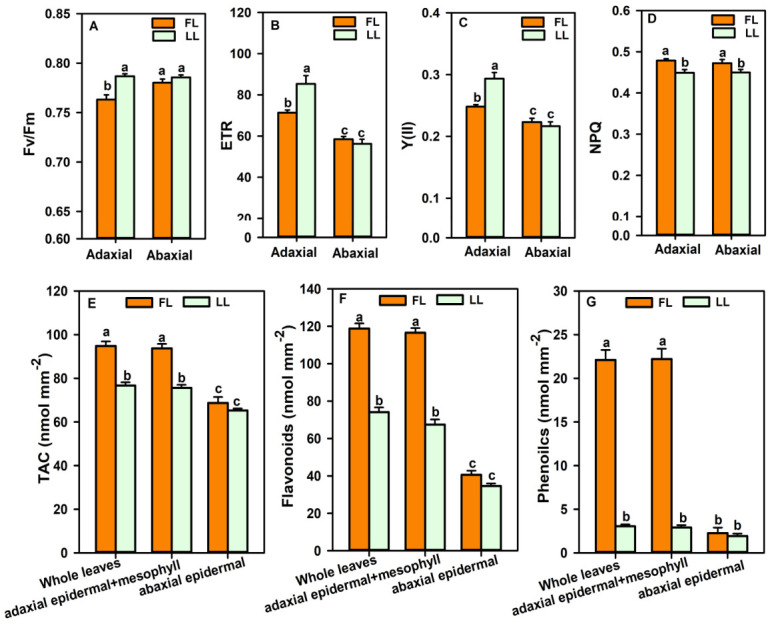
Changes in chlorophyll fluorescence parameters and leaf antioxidant content in *T. spathacea* under FL and LL conditions. (**A**–**D**) show the maximum photochemical efficiency of photosystem II (PSII) (F_v_/F_m_), the electron transport rate (ETR), the effective photochemical quantum yield of PSII (ΦPSII or Y(II)), and non-photochemical quenching (NPQ) on the adaxial and abaxial leaf surfaces under FL and LL, and (**E**–**G**) present the total antioxidant capacity, flavonoid content, and total phenolic content in intact leaf disks, samples containing only the adaxial epidermis and mesophyll cells, and samples containing only the abaxial epidermis under FL and LL. All data are presented as mean ± standard deviation (*n* = 5). Different letters above bars indicate statistical significance (*p* < 0.05).

Under FL and LL treatments, the chlorophyll fluorescence parameters of *T. spathacea* showed significant differences. For the abaxial side with anthocyanin accumulation, there was no significant difference in the maximum photochemical efficiency of PSII (F_v_/F_m_) between FL and LL conditions. In contrast, for the adaxial side without anthocyanin accumulation, the F_v_/F_m_ value under LL was significantly higher than that under FL (Figure 3A). The electron transport rate (ETR) and the effective photochemical quantum yield of PSII (Y(II)) results showed that the adaxial side without anthocyanin accumulation under LL exhibited the highest ETR and Y(II), followed by the adaxial side without anthocyanin accumulation under FL, while the abaxial side with anthocyanin accumulation showed the lowest ETR and Y(II) (Figure 3B,C). Whether it is in the abaxial or adaxial side, the NPQ of the leaves under FL is significantly higher than that under LL (Figure 3D). Total antioxidant capacity (TAC) under FL was significantly higher than under LL in both intact leaf disks and adaxial epidermis + mesophyll cells samples, whereas no significant difference in TAC was observed between these two sample types under the same light conditions. For the abaxial epidermal layer, TAC showed no significant difference between FL and LL, and its values were markedly lower than those of intact leaf disks and adaxial epidermis + mesophyll samples (Figure 3E). The trends for flavonoid and total phenolic contents were consistent with those observed for TAC (Figure 3F,G).

### 2.4. The Content of Photosynthetic Pigments in Leaves of Purple Abaxial Evergreen Under FL and LL Environments

**Figure 4:**
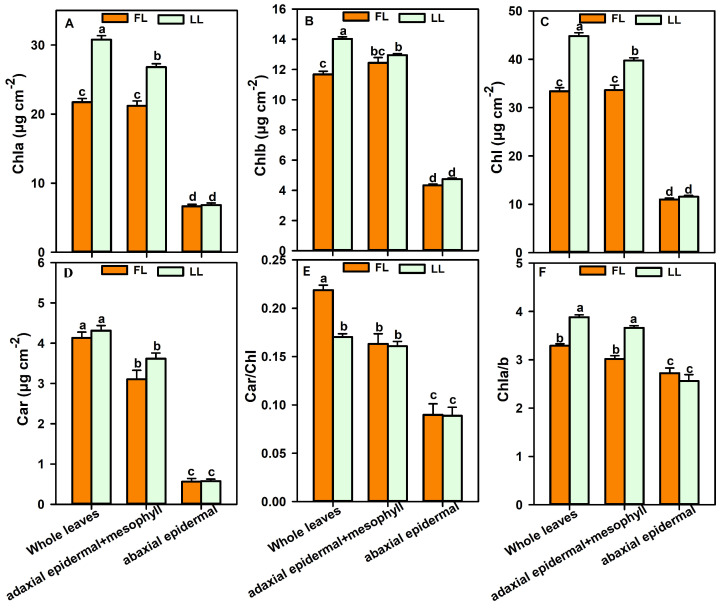
Changes in photosynthetic pigment contents in *T. spathacea* leaves under FL and LL conditions. (**A**–**F**) show the photosynthetic pigment contents of intact leaf disks, samples consisting only of the adaxial epidermal layer + mesophyll cells, and samples consisting only of the abaxial epidermal layer under FL and LL conditions, respectively. All data are presented as means ± standard deviation (*n* = 5). Different letters above bars indicate statistical significance (*p* < 0.05).

Under FL and LL treatments, the photosynthetic pigment contents of *T. spathacea* exhibited significant differences. Under FL conditions, there was no significant difference in Chl a content between intact leaf disks and samples consisting only of the adaxial epidermal layer + mesophyll cells; however, both were significantly lower than under LL conditions. Under LL conditions, the Chl a content of intact leaf disks was significantly higher than that of samples consisting only of the adaxial epidermal layer + mesophyll cells. For samples consisting only of the abaxial epidermal layer, Chl a content showed no significant difference between FL and LL, but was significantly lower than that of intact leaf disks and samples consisting of the adaxial epidermal layer + mesophyll cells (Figure 4A). The trends for Chl b and total Chl contents were consistent with those of Chl a (Figure 4B,C). The Car results showed that intact leaf disks had the highest content, followed by samples consisting of the adaxial epidermal layer + mesophyll cells, and samples consisting only of the abaxial epidermal layer had the lowest, with no relationship to light treatment (Figure 4D). Regarding the Car/Chl ratio, under FL conditions, intact leaf disks had a significantly higher Car/Chl ratio than under LL conditions. For samples consisting of the adaxial epidermal layer + mesophyll cells and samples consisting only of the abaxial epidermal layer, there was no significant difference in Car/Chl between FL and LL, but the adaxial epidermal layer + mesophyll cells samples had a significantly higher ratio (Figure 4E). The Chl a/b ratio results showed that under FL conditions, intact leaf disks and samples consisting of the adaxial epidermal layer + mesophyll cells had the highest Chl a/b ratio, followed by the same sample types under LL conditions, while samples consisting only of the abaxial epidermal layer had the lowest Chl a/b ratio under both FL and LL conditions (Figure 4F).

### 2.5. Comparative Analysis of Differentially Expressed Genes

**Figure 5:**
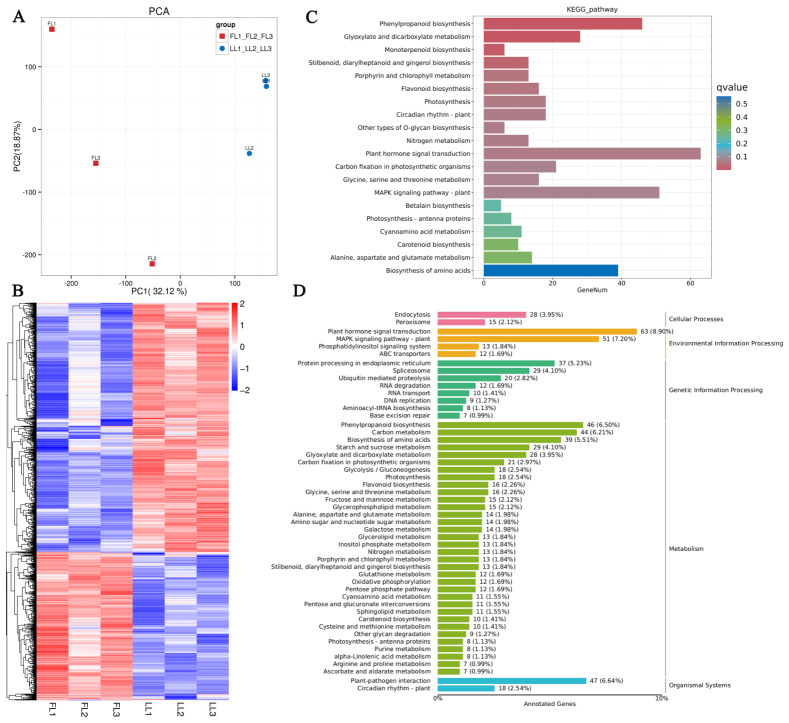
Transcriptomic data analysis of *T. spathacea* leaves under FL and LL conditions (*n* = 3). (**A**) Principal component analysis (PCA) score plot of sequencing samples under FL and LL conditions; (**B**) heatmap clustering of differentially expressed genes (DEGs) across all samples under FL and LL conditions; (**C**) number of differentially expressed genes in KEGG pathways under FL and LL conditions; and (**D**) KEGG classification of differentially expressed genes under FL and LL conditions.

Prior to transcriptome sequencing analysis, multivariate statistical analysis was performed on *T. spathacea* samples under FL and LL conditions using principal component analysis (PCA). The results showed high reproducibility among samples, indicating high reliability of the sequencing data and permitting further analysis (Figure 5A). Hierarchical clustering analysis was conducted on the combined set of differentially expressed genes (DEGs) from all samples under FL and LL conditions, and clustering heatmaps were generated for each differential group (Figure 5B). Under LL conditions, the number of upregulated DEGs was greater than that under FL conditions, although FL conditions also exhibited a considerable number of upregulated genes. In KEGG pathway analysis, 20 metabolic pathways with the highest numbers of DEGs were selected. To further determine the functions of the DEGs, it was found that metabolic pathways such as those involved in photosynthesis, photosynthetic pigments, and flavonoids were significantly enriched (Figure 5C). Additionally, in the FL vs LL comparison, the majority of DEGs were annotated to 50 KEGG pathways (Figure 5D).

### 2.6. Differentially Expressed Genes Involved in Anthocyanin Biosynthesis

**Figure 6:**
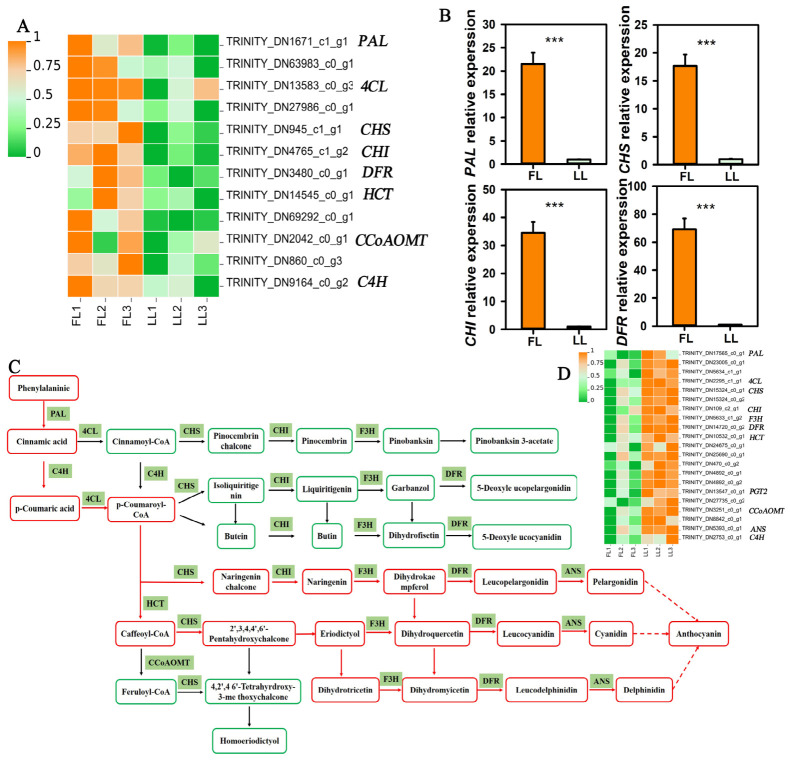
Expression analysis of anthocyanin-related genes in *T. spathacea* leaves under FL and LL conditions. (**A**,**D**) Heatmap showing the expression levels of genes involved in the anthocyanin biosynthesis pathway in *T. spathacea* leaves under FL and LL conditions. (**B**) Validation of the expression levels of anthocyanin biosynthesis pathway-related genes by real-time PCR under FL and LL conditions. (**C**) Schematic diagram of the anthocyanin biosynthetic pathway in *T. spathacea* leaves. *** indicates significant difference at the 0.001 level.

Transcriptomic analysis revealed that the differentially expressed genes involved in anthocyanin biosynthesis in leaves under FL and LL conditions included *PAL*, *4CL*, *CHS*, *CHI*, *DFR*, *HCT*, *CCoAOMT*, and *C4H*. The results showed that all of these genes were significantly upregulated under FL (Figure 6A). To validate the reliability of the RNA-seq data, four genes related to anthocyanin biosynthesis were selected for qRT-PCR. The expression patterns obtained from qRT-PCR were consistent with those from RNA-seq (Figure 6B), confirming that the transcriptomic data are reliable and suitable for further investigation of anthocyanin biosynthesis in *T. spathacea* leaves under different light environments. In addition, we identified several anthocyanin biosynthesis-related genes that were significantly downregulated under FL conditions (Figure 6D). By constructing a schematic diagram of the anthocyanin biosynthetic pathway (red boxes) alongside genes involved in both anthocyanin biosynthesis and the synthesis of other compounds, such as pinobanksin 3-acetate, 5-deoxyle ucopelargonidin, and 5-deoxyle ucocyanidin (green boxes), we uncovered the possible reasons why the expression levels of some anthocyanin biosynthesis-related genes were significantly increased under LL conditions (Figure 6C).

### 2.7. Differentially Expressed Genes Related to Photosynthetic Metabolic Pathways

**Figure 7:**
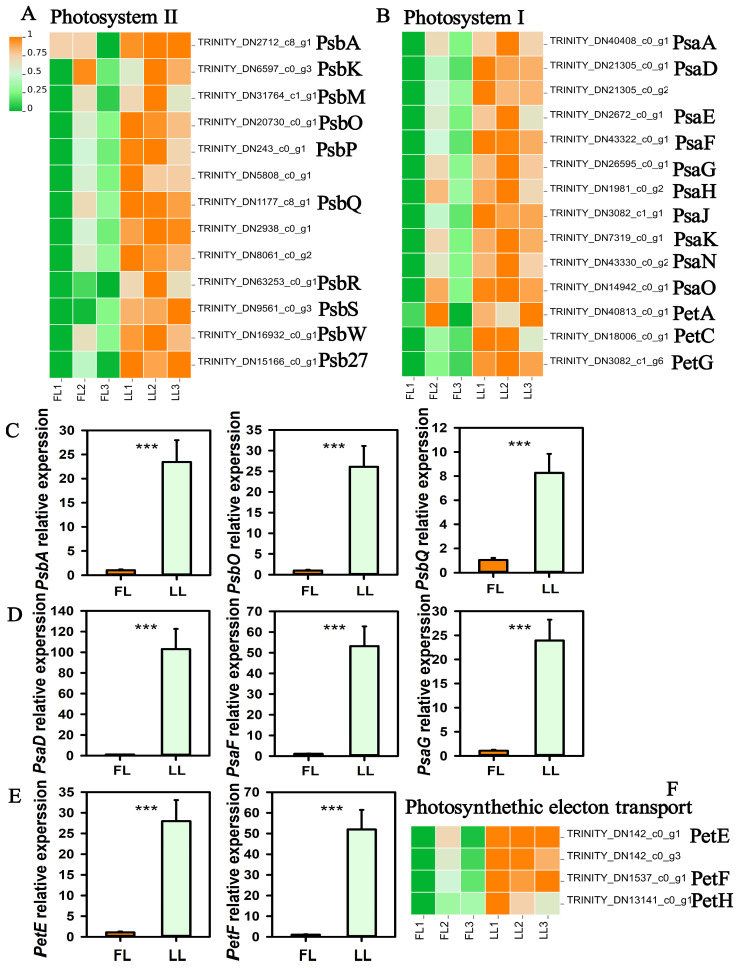
Expression analysis of genes related to photosynthetic metabolic pathways in *T. spathacea* leaves under FL and LL conditions. (**A**,**B**,**F**) Heatmaps showing the expression levels of genes associated with photosystem II, photosystem I, and photosynthetic electron transport in *T. spathacea* leaves under FL and LL conditions. (**C**–**E**) Validation of the expression levels of photosynthetic pathway-related genes by real-time PCR under FL and LL conditions. *** indicates significant difference at the 0.001 level.

Furthermore, as part of the transcriptome analysis, we also screened for metabolic pathways related to photosynthesis, including genes associated with photosystem II (PSII), photosystem I (PSI), and photosynthetic electron transport, and performed heatmaps to visualize their expression patterns. The results showed that, under LL conditions, the expression levels of *PsbA*, *PsbK*, *PsbM*, *PsbO*, *PsbP*, *PsbQ*, *PsbR*, *PsbS*, *PsbW*, and *Psb27* from PSII (Figure 7A), *PsaA*, *PsaD*, *PsaE*, *PsaF*, *PsaG*, *PsaH*, *PsaJ*, *PsaK*, *PsaN*, *PsaO*, *PetA*, *PetC*, and *PetG* from PSI (Figure 7B), as well as *PetE*, *PetF*, and *PetH* involved in photosynthetic electron transport (Figure 7F), were all significantly higher than under FL conditions. To validate the RNA-seq data, we selected three genes from PSII, three from PSI, and two related to photosynthetic electron transport for qRT-PCR. The qRT-PCR results confirmed the RNA-seq findings, showing similar expression patterns (Figure 7C–E), thereby confirming the reliability of the transcriptome data.

## 3. Discussion

This study systematically characterized the morphological, biomass, physiological, and molecular responses of *T. spathacea* under different light intensities, and for the first time elucidated its light adaptation strategy from the perspective of dorsiventral functional specialization of leaves and the anthocyanin-mediated trade-off between photoprotection and photosynthesis. Under FL conditions, the plants prioritized survival by enriching anthocyanins in the abaxial side, increasing leaf thickness, adopting a more erect leaf angle, and enhancing thermal dissipation and antioxidant capacity, thereby establishing a multilayered photoprotective system. Under LL conditions, the plants shifted to a growth priority strategy, upregulating photosynthesis-related genes, increasing chlorophyll content and electron transport efficiency, and enhancing light utilization and carbon assimilation. The photoprotection photosynthesis trade-off model established in this study provides new physiological and molecular evidence for understanding the adaptive mechanisms of shade-tolerant plants to heterogeneous light environments.

### 3.1. Reconfiguration of Leaf Morphology Under FL and Precise Localization of Anthocyanins

Morphological plasticity represents the primary adaptive strategy of plants in response to changes in light conditions, and the magnitude of such adjustments is often closely related to a species ecological niche breadth [32,33]. This study found that under FL conditions, leaf thickness of *T. spathacea* increased significantly (Figure 2A), while the angle between newly matured leaves and the ground increased to approximately 80° (Figure 1C,D and Figure 2D,E), indicating a more upright leaf orientation. In contrast, under LL conditions, leaves were thinner (Figure 2A), with both leaf width and length significantly greater than those in the FL group (Figure 2B,C). Moreover, leaves under LL were more horizontally oriented, forming an angle of approximately 40° with the ground (Figure 2E). The increase in leaf thickness under FL is beneficial for establishing a longer light absorption path within the leaf interior and provides additional cellular space for the accumulation of photoprotective compounds such as anthocyanins [34]. Adjustments in leaf angle directly alter the effective incident light intensity on the leaf surface: under FL, upright leaves increase the angle between the leaf blade and incoming solar radiation, significantly reducing the photon flux density incident per unit leaf area, thereby alleviating excitation pressure on PSII reaction centers [35]. Under LL, horizontally oriented leaves maximize interception of diffuse light from above, thereby improving light capture efficiency [22,23,24]. Under FL conditions, key genes involved in anthocyanin biosynthesis (e.g., *PAL*, *4CL*, *CHS*, *CHI*, *DFR*) were significantly upregulated (Figure 6A), which was highly consistent with the physiological phenotype of increased anthocyanin content observed under FL. However, it is worth noting that some genes related to anthocyanin synthesis (such as those involved in the bypass metabolism of pinobanksin 3-acetate, 5-deoxyle ucopelargonidin, and 5-deoxyle ucocyanidin) were instead downregulated under the FL condition (Figure 6D). This differential expression pattern reveals a targeted redistribution of carbon flux among distinct biosynthetic branches under high light, whereby the plant preferentially channels the carbon skeleton into high-efficiency anthocyanin synthesis rather than into other flavonoid derivatives. Such fine-scale transcriptional regulation provides a molecular explanation for how *T. spathacea* achieves precise photoprotection under conditions of limited resource availability.

### 3.2. Distribution Characteristics of Abaxial Side Anthocyanins and Their Multiple Photoprotective Functions

The specific accumulation of anthocyanins in the abaxial epidermis of *T. spathacea* leaves represents a distinctive distribution pattern with special morphological and ecological adaptive value. Its functions can be inferred from three aspects. First, this structure can filter high-energy blue-violet light from reflected environmental light [36,37]. This species is native to forest edges and shaded understory habitats, where the abaxial side primarily receives reflected and diffuse light from the soil and underlying vegetation. Such light has a high proportion of blue-violet wavelengths [36], which can easily penetrate the leaf and damage mesophyll cells. Anthocyanins in the abaxial epidermis absorb blue-violet light in the 400–500 nm range, reducing the light energy input into chloroplasts and thereby suppressing reactive oxygen species generation [38]. Unlike in most plants, anthocyanins in this species are distributed exclusively on the abaxial side, representing a unique adaptive strategy evolved specifically for the reflected light environment of the understory [12,39]. Second, the ventral–abaxial compartmentalization enables spatial decoupling of light capture from antioxidant defense. If anthocyanins were distributed on the adaxial side, they would reduce the transmittance of photosynthetically active radiation and directly inhibit carbon assimilation [40]. By contrast, the distribution of anthocyanins on the abaxial side allows the adaxial side to efficiently absorb the light radiation required for photosynthesis, thus ensuring the normal operation of photosynthetic processes, while the abaxial side anthocyanin layer intercepts harmful transmitted light. This configuration defends against photo-oxidative stress while maximizing the plant’s growth capacity under LL conditions, serving as the core structural basis for its combined shade-tolerance and FL adaptability. Third, anthocyanin accumulation and upright leaf angles form a synergistic photoprotective system. Under FL, the angle between the leaves and the ground increases, and the leaves become more upright, exposing the accumulated anthocyanins on the abaxial epidermis to incident light. This not only reduces the amount of direct light intercepted through leaf angle adjustment, but also filters the transmitted light through the abaxial epidermal anthocyanins. Together, these mechanisms reduce light energy input, thereby maintaining PSII functional stability even when photosynthetic rates are downregulated, while directing metabolic energy toward antioxidant synthesis and photodamage repair. Considering that the biomass per plant under LL conditions was significantly higher than that under FL conditions (Figure 1H), these findings indicate that *T. spathacea* achieves a dynamic balance between growth and survival under different light environments through coordinated regulation of morphological and biochemical mechanisms.

### 3.3. Synergistic Regulation of Photochemical Efficiency, Photosynthetic Capacity, Antioxidants, and Energy Dissipation Under FL Stress

Chlorophyll fluorescence parameters can intuitively reflect the function of photosystem II (PSII) and light-use efficiency in plants [3]. Our results show that under FL conditions, the F_v_/F_m_ in the without-anthocyanin adaxial side of *T. spathacea* was significantly lower than that under LL. In contrast, no significant difference in F_v_/F_m_ was observed between the two light conditions for the with-anthocyanin adaxial side. This indicates that FL primarily induces photoinhibition of PSII in the light-exposed adaxial side, while anthocyanins on the abaxial side attenuate the impact of FL on the photosynthetic apparatus. Furthermore, under FL, the abaxial side maintained a relatively high F_v_/F_m_, and samples consisting only of the abaxial epidermal layer exhibited weak TAC and low levels of antioxidant compounds (Figure 3E–G). This supports the conclusion that anthocyanin accumulation on the abaxial side primarily functions as an optical filter, reducing excessive light energy entering photosynthetic tissues and mitigating damage to photosynthetic proteins by reactive oxygen species, thereby protecting the structural integrity of the PSII reaction center [30]. Gas exchange measurements revealed that Pn and Gs in the FL group were significantly lower than those in the LL group, while Rd and Tr were significantly higher, indicating that high light markedly suppresses photosynthetic carbon assimilation capacity. This is highly consistent with overall plant growth performance: the biomass per plant under LL was significantly greater than that under FL, demonstrating that the higher photosynthetic carbon assimilation efficiency under low light provides a sufficient material basis for biomass accumulation, whereas the suppression of photosynthetic capacity under high light directly limits plant growth potential. Additionally, the leaf ETR decreased significantly under FL, especially in the without-anthocyanin adaxial side, where excitation energy could not be efficiently converted into chemical energy, further exacerbating the risk of photo-oxidation. Although the abaxial surface showed the lowest ETR under FL, this seemingly paradoxical observation reflects an adaptive trade-off: with light filtering by anthocyanins as its primary role, the abaxial surface prioritizes reducing light intensity reaching the mesophyll and alleviating PSII excitation pressure, thereby actively downregulating electron transport efficiency. This strategy of sacrificing some photosynthetic capacity to maintain photosystem stability represents a typical survival strategy of *T. spathacea* under FL. Transcriptomic data provided molecular evidence supporting the adaptive regulation of photosynthetic function. Under LL, genes associated with photosystem I, photosystem II, and the photosynthetic electron transport chain (e.g., *PsbA*, *PsaA*, *PetE*) were significantly upregulated, which corresponded well with the higher Pn, chlorophyll content, and ETR observed under LL. This suggests that under LL, the plant comprehensively enhances photosynthetic structure and function through transcriptional reprogramming of gene expression. In contrast, under FL, the expression of photosynthesis-related genes was suppressed, consistent with the physiological phenotype of reduced photosynthetic capacity, thereby fully reflecting the metabolic trade-off in which plant survival takes precedence over growth.

The significantly increased Rd observed in the FL group represents an important metabolic compensatory mechanism. On one hand, dark respiration consumes photosynthetically fixed carbohydrates, alleviating feedback inhibition caused by constrained carbon assimilation and maintaining carbon metabolic balance [41]. On the other hand, it may provide precursors for secondary metabolism and sustain cellular carbon homeostasis [42]. Compared with LL conditions, Gs was significantly reduced under FL, which is likely a passive adaptive response to mitigate excessive leaf water loss through active stomatal closure or reduced stomatal aperture [43]. In the antioxidant defense system, TAC, flavonoid, and phenolic content in both intact leaves and adaxial epidermal layer + mesophyll tissues were significantly higher under high light than under LL, whereas no differences between light treatments were detected in the abaxial epidermal layer containing anthocyanins. This indicates that the high-light-induced non-enzymatic antioxidant response occurs primarily in the mesophyll cells, which serve as the main site of photosynthesis and the primary site of ROS production [44]. The substantial accumulation of antioxidant compounds therein enables timely scavenging of free radicals, thereby alleviating oxidative damage to PSII, the D1 protein, and thylakoid membranes [17,18]. The increased Car/Chl under FL, together with the enhanced NPQ, indicates that thermal dissipation pathways are fully activated [45]. Carotenoids are core components of the xanthophyll cycle. Under FL, violaxanthin is converted to zeaxanthin, which dissipates excess light energy as heat, stabilizes the light-harvesting complex structure, and reduces ROS generation [13,14,15]. Concurrently, the decreased chlorophyll content and increased carotenoid content represent an important adaptive strategy by which the plant proactively downregulates its light capture capacity to avoid light energy overload.

## 4. Materials and Methods

### 4.1. Plant Materials

This experiment was conducted from July to September 2023 at the Biological Garden (23.656574° N, 116.665756° E) of Hanshan Normal University, Guangdong Province. The experimental site features a southern subtropical monsoon maritime climate, with an average summer temperature of approximately 31 °C, and an average winter temperature of about 16 °C. The mean annual precipitation is 1769.4 mm, and the annual average relative humidity is 77%. Seasonal differences in light conditions are pronounced: at noon on clear summer days, the photosynthetically active radiation (PAR) ranges from 1800 to 2200 μmol m^−2^ s^−1^, while on clear winter days, PAR ranges from 1000 to 1300 μmol m^−2^ s^−1^. Two light intensity treatments were established: full sunlight (100% natural light, FL) and low light (30% natural light, LL). The low-light treatment was achieved using double-layer shade nets, and the actual light intensity was calibrated using a quantum photometer. All plants were maintained under uniform cultivation management: regular watering was performed each evening, and compound fertilizer was applied once a month at a rate of 0.5 g per plant. The soil in the experimental field is sandy loam with high organic matter content, loose texture, and good aeration and drainage properties. After two years of acclimatization to the respective light conditions, the plants were used for subsequent measurements of various traits.

### 4.2. Measurement of Growth Traits

The experiment was conducted at 9:00 a.m. within the plant cultivation area. Healthy and robust *T. spathacea* plants at the same growth stage, exhibiting good vigor, were selected from a uniformly cultivated population. All selected plants were free from visible pests, diseases, or mechanical damage. To minimize the influence of individual developmental differences on the results, the measurements were consistently targeted at the third and fourth pairs of fully expanded mature functional leaves from the top of the plant, i.e., the left and right symmetrical leaves. Leaf length (measured from the leaf base at the stem–leaf junction to the leaf tip) and leaf width (the maximum distance between the left and right margins at the middle part of the leaf) were measured using a standard ruler (1 mm resolution). Leaf thickness was measured at the central region of the same leaf using a vernier caliper (resolution: 0.02 mm). Eleven biological replicates were performed.

### 4.3. Leaf Angle Relative to Ground

Before measurement, upright plants were selected to ensure that the main stem (apical shoot segment) was completely perpendicular to the ground, thereby ensuring consistency of the reference plane (the ground) for all angular measurements. Leaf angles were measured manually using a precision protractor (resolution: 1°). The measured angle was defined as the angle between the main extension plane of the target leaf and the horizontal ground surface. Specifically, the center point of the protractor was aligned with the junction of the leaf base and the stem, the stationary arm was kept horizontal (parallel to the ground), and the movable arm was extended and aligned along the midrib direction of the leaf; the indicated angle value was then recorded. Measurements were taken separately for the left and right target leaves of each plant. If a leaf exhibited natural curvature, the orientation of the midrib at the central region of the leaf was used as the reference. All measurements were performed consecutively within a short period under identical environmental conditions by the same experimenter to minimize inter-operator errors and deviations caused by temporal fluctuations. Nineteen biological replicates were performed.

### 4.4. Epidermal Tissue Separation and Sample Preparation

Fresh, healthy, mature functional leaves without visible disease symptoms were collected from the experimental plants. After sampling, the leaves were gently rinsed with deionized water to remove dust and attached debris, and then carefully blotted dry with clean filter paper. Leaf disks, each 6 mm in diameter, were punched from areas of the same leaf age on both sides of the midrib using a hole puncher. Care was taken to avoid the main vein and major lateral veins to ensure that the sampled tissues were primarily composed of mesophyll and epidermis, maintaining textural uniformity. To compare the photosynthetic or physiological characteristics of different tissue structures in the leaves of *T. spathacea,* a mechanical separation method was used to obtain experimental materials. The separation was performed under a stereomicroscope (SMZ-140, Motic, Xiamen, China). Using a sharp single-edged blade or a fine dissection knife, the leaf disks were carefully dissected to obtain two types of tissue samples: those consisting only of the adaxial epidermal layer + mesophyll cells, and those consisting only of the abaxial epidermal layer. All dissection procedures were performed rapidly, and the separated samples were immediately transferred into phosphate buffer (pH = 7.0) to minimize tissue damage and loss of physiological activity. Consequently, three types of experimental materials were obtained: (1) intact, non-separated leaf disks, (2) disks containing the adaxial epidermis + mesophyll cells, and (3) disks containing the abaxial epidermal layer. Five replicates were set for each experimental group. Five biological replicates were performed.

### 4.5. Anthocyanin Extraction and Quantification

Anthocyanins were extracted and quantified using a 1% hydrochloric acid-methanol solution [17]. For each of the three types of samples prepared as described above, two 6 mm leaf disks were placed into a pre-prepared centrifuge tube containing 0.6 mL of 1% (*v*/*v*) HCl-methanol. The tubes were then incubated in the dark at 4 °C for 36 h to ensure complete extraction of anthocyanins. Following the extraction, 0.6 mL of chloroform and 0.3 mL of distilled water were sequentially added to each tube. The mixtures were thoroughly vortexed and then allowed to settle. This resulted in the separation of the solution into two phases: anthocyanins partitioned into the adaxial methanol-aqueous phase, while chlorophyll partitioned into the lower chloroform phase. The adaxial phase was carefully aspirated, and its absorbance was measured at 530 nm using a UV-2450 spectrophotometer (Shimadzu, Tokyo, Japan). Anthocyanin content was calculated using a standard curve established with known concentrations of cyanidin-3-O-glucoside (Jin Clone, Biotechnology Co., Ltd., Beijing, China). Five biological replicates were performed.

### 4.6. Determination and Extraction of Photosynthetic Pigments

Photosynthetic pigments in the leaves were extracted and determined using the 80% acetone immersion spectrophotometric method. For each of the three prepared sample types, two leaf disks (6 mm in diameter) were placed into pre-prepared 2 mL centrifuge tubes, to which 2 mL of 80% acetone solution was added. All samples were incubated in darkness at 4 °C for 48 h. During this period, the tubes were manually shaken or vortexed every 8 h to ensure complete and uniform pigment extraction. Extraction was considered complete when the leaf tissue became completely decolorized (white), indicating that chlorophyll had been fully extracted and was ready for quantification. The absorbance of the chlorophyll extract was measured at 663, 645, and 470 nm using a UV2450 spectrophotometer (Shimadzu, Tokyo, Japan), with 80% acetone serving as the blank control. The contents of chlorophyll a (Chl a), chlorophyll b (Chl b), total chlorophyll (Chl), and carotenoids (Car) were calculated according to the formulas of Wellburn [46]. Chl a (μg mL^−1^) = 12.21A663 − 2.81A646; Chl b (μg mL^−1^) = 20.13A646 − 5.03A663; Chl (μg mL^−1^) = Chl a + Chl b; Car (μg mL^−1^) = (1000A470 − 3.27Chl a − 104Chl b)/198. Five biological replicates were performed.

### 4.7. Chlorophyll Fluorescence Measurement

Chlorophyll fluorescence parameters were measured using a PAM2500 portable modulated chlorophyll fluorometer (Waltz, Effeltrich, Germany). For each experimental treatment, five healthy, lesion-free leaves were randomly selected for replicated measurements. Leaves were first dark-adapted for 30 min to ensure a stable physiological state. The adaxial side was then positioned facing the measuring window of the fluorometer. Under complete dark conditions, the minimal fluorescence yield (F_0_) was recorded, followed by the application of a 10 s saturating pulse (9000 μmol m^−2^ s^−1^) to determine the maximal fluorescence yield (F_m_). Subsequently, the actinic light intensity was set to 800 μmol m^−2^ s^−1^ to obtain steady-state chlorophyll fluorescence. The PamWin-3 software automatically calculated and recorded relevant chlorophyll fluorescence parameters, including the maximum photochemical efficiency of PSII (F_v_/F_m_), the effective photochemical quantum yield of PSII (ΦPSII or Y(II)), the ETR, and the NPQ. After completing the measurement on the adaxial side, the same leaf was again dark-adapted for 30 min, then flipped over so that the abaxial side faced the measuring window, and the entire measurement procedure was repeated to obtain the corresponding fluorescence parameter dataset for the abaxial side. Five biological replicates were performed.

### 4.8. Extraction and Determination of Flavonoids, Phenolics, and TAC

Flavonoids, phenolic compounds, and TAC are all extracted using 95% methanol. Three types of prepared samples (intact leaf disks, samples consisting of the adaxial epidermis + mesophyll cells, and samples of the abaxial epidermis containing anthocyanins) were used. Two leaf discs from each sample type were placed into pre-prepared centrifuge tubes containing 1.5 mL of 95% methanol and incubated at 4 °C in darkness for 48 h. Flavonoid content was determined according to the method described by Heimler et al. [47]. Phenolic content was measured using the Folin–Ciocalteu method, optimized based on the protocol of Ainsworth and Gillespie [48]. TAC was assessed by the DPPH radical scavenging assay following the method of Saha et al. [49]. Five biological replicates were performed.

### 4.9. Gas Exchange Measurements

Leaf photosynthetic capacity was measured using a portable photosynthesis measurement system (Li-6400XT, Li-COR Inc., Lincoln, NE, USA) equipped with a standard red-blue light source leaf chamber. Measurements were performed following the method described by Zhang et al. [17] with minor modifications. The measurement conditions were set as follows: photosynthetically active radiation of 800 µmol m^−2^ s^−1^ (provided by the built-in LED light source with a red-to-blue light ratio of 9:1), leaf chamber temperature of 25 ± 0.5 °C, relative humidity of 60 ± 5%, and CO_2_ concentration stabilized at 400 µmol mol^−1^ using the system CO_2_ injection unit. For each treatment, five mature, healthy leaves were randomly selected for measurement. Before recording, leaves were light-induced at the set light intensity for 5–10 min until gas exchange parameters reached a steady state, after which the Pn, Tr, Gs, and Rd, respectively, were recorded. All measurements were conducted at the same time each day to minimize diurnal variation. Five biological replicates were performed.

### 4.10. Transcriptomic Analysis

Total RNA was extracted from each sample using the Total RNA Extractor (Trizol) Kit (B511311, Sangon Biotech, Shanghai, China) following the manufacturer’s instructions. Libraries were constructed from total RNA samples extracted from leaves subjected to FL and LL treatments using the Biomarker RNA Library Preparation Kit. (Biomarker Technologies, Beijing, China) RNA purity and concentration were assessed using a NanoDrop 2000 spectrophotometer (Thermo Fisher Scientific, Waltham, MA, USA), and RNA integrity was accurately evaluated using an Agilent 2100/LabChip GX system (Agilent Technologies, Santa Clara, CA, USA). After the sample is tested and found to be qualified, the database will be established. Following database preparation, initial quantification was conducted using a Qubit 3.0 fluorometer, requiring a concentration of at least 1 ng/µL. Subsequently, the insert fragment size was assessed using the Qsep400 high-throughput analysis system (Bioptic Inc., New Taipei, Taiwan, China). Once the insert fragment size met expectations, the effective concentration of the library (>2 nM) was accurately quantified using Q-PCR (Bio-Rad, Hercules, CA, USA) to ensure library quality. After passing quality control, the libraries were sequenced using a high-throughput sequencing platform in PE150 mode. After obtaining high-quality sequencing data, sequence assembly was performed. Clean reads were assembled de novo into transcripts using Trinity. To ensure the reliability of transcriptome data, the quality of the sequencing libraries was evaluated through randomness tests, fragment length distribution analysis, and saturation analysis. Unigene sequences were aligned against the NR, Swiss-Prot, COG, KOG, eggNOG 4.5, and KEGG databases using DIAMOND v4.6.8 software (Crystal Impact GbR, Bonn, Germany). KEGG Orthology results for Unigenes were obtained using KOBAS (KEGG Orthology Based Annotation System, Peking University, Beijing, China). InterProScan (EMBL-European Bioinformatics Institute, Hinxton, Cambridge, UK) was used to analyze GO Orthology results for new genes based on the InterPro (EMBL-EBI, Cambridge, United Kingdom) integrated database. After predicting the amino acid sequences of Unigenes, HMMER v5.7.0 software (Eddy/Rivas Laboratory, HHMI, Ashburn, VA, USA) was used to align them against the Pfam database to obtain annotation information. Coding region sequences and corresponding amino acid sequences of Unigenes were predicted using TransDecoder v5.7.1 software (Cambridge, MA, USA). Reads obtained from sequencing were aligned against the Unigene library using Bowtie v2.5.1, and expression levels were estimated using RSEM v1.3.3. The expression abundance of each Unigene was represented by the FPKM value. Differentially expressed genes were subjected to functional annotation against the databases. Identify differentially expressed genes (DEGs) between two samples using DESeq2 v1.30.0 software (Biomarker Technologies, Beijing, China) based on the Count values of genes in each sample. In the process of detecting differentially expressed genes, fold change ≥ 2 and q-value < 0.001 are used as screening criteria. The transcriptome data are presented in the Supplementary Materials. Three biological replicates were performed.

### 4.11. Gene Expression Analysis

The total RNA was extracted from leaf samples using TRIzol reagent (Invitrogen, Waltham, MA, USA) following the manufacturer’s instructions. Complementary DNA was synthesized using TopScript™ RT DryMIX (dT18) (Enzynomic, Daejeon, Republic of Korea) according to the manufacturer’s protocol. Quantitative reverse transcription polymerase chain reaction analysis was performed using the SYBR Premix Ex Taq™ II kit (Takara, Tokyo, Japan) in conjunction with a Bio-Rad CFX96 real-time PCR system (CFX96, Bio-Rad, Hercules, CA, USA). The cycling conditions were as follows: initial denaturation at 95 °C for 30 s, followed by 39 cycles of 95 °C for 5 s and 60 °C for 34 s, and finally a melt curve analysis from 65 °C to 95 °C. GAPDH was used as an internal reference gene. Primers used for qRT-PCR analysis are listed in Table 1. Gene expression levels were analyzed using the 2^−ΔΔCt^ method [50]. Three biological replicates were performed.

### 4.12. Statistical Analysis

Raw data were initially processed using Microsoft Excel 2016. All subsequent statistical analyses were performed using SPSS Statistics 19.0 (IBM, Armonk, NY, USA). Differences between treatments were evaluated using independent sample *t*-tests, and one-way analysis of variance (ANOVA) followed by Duncan’s multiple range test was conducted to compare means among treatments, in order to determine the significance of differences in physiological parameters of *T. spathacea* under different light conditions. The significance of the analysis was set at the level of 0.05. Before analysis, the Shapiro–Wilk test checked data normality and the Levene test assessed data homogeneity. Regression analyses and graphical representations were carried out using SigmaPlot 14 (Systat Software Inc., Richmond, VA, USA) and GraphPad Prism 10 (Boston, MA, USA). All data are presented as mean ± standard error (SE). Different lowercase letters indicate significant differences between the control treatments. * indicates a significant difference at the 0.05 level; ** indicates a significant difference at the 0.01 level; and *** indicates a significant difference at the 0.001 level.

## 5. Conclusions

**Figure 8:**
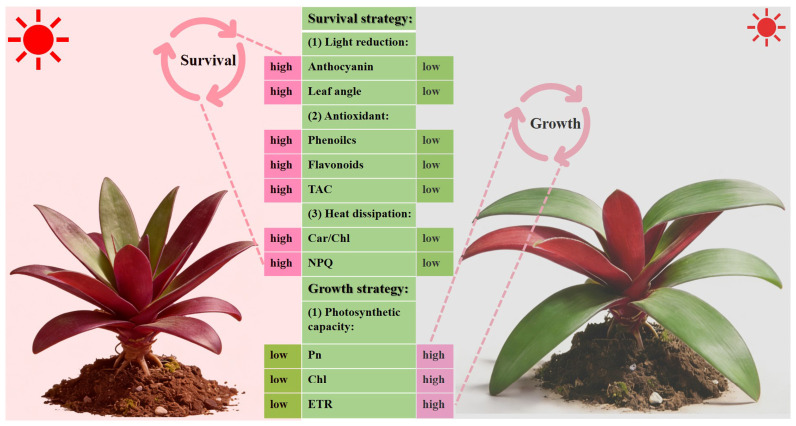
Schematic diagram of the trade-off between survival and growth strategies in *T. spathacea* under FL and LL conditions. Left (FL): Plants prioritize survival through three mechanisms: (1) light reduction (increased anthocyanin accumulation, adjustment of leaf angle), (2) antioxidant defense (enhanced phenolics, flavonoids, total antioxidant capacity, TAC), and (3) thermal dissipation (increased carotenoid/chlorophyll (Car/Chl) ratio, non-photochemical quenching, NPQ). Right (LL): Plants shift toward growth by enhancing photosynthetic capacity (higher net photosynthetic rate (Pn), chlorophyll content (Chl), and electron transport rate (ETR)). Pink represents FL growth (left), and gray indicates growth in LL areas (right).

This study systematically elucidated the adaptation mechanisms of *T. spathacea* to varying light intensities by integrating morphological observations, physiological measurements, chlorophyll fluorescence analysis, gas exchange measurements, and transcriptomic techniques. This species achieves broad-spectrum adaptation to both FL and LL environments through the functional specialization of the adaxial and abaxial sides, as well as through anthocyanin-mediated photoprotection and a trade-off between photoprotection and photosynthesis. Under FL conditions, the plant adopts a survival-first strategy: genes involved in anthocyanin biosynthesis are upregulated, and anthocyanins are specifically enriched in the abaxial side. Concurrently, leaves become thicker and more upright, with the anthocyanin filtering out harmful blue and ultraviolet light. The plant also enhances non-photochemical quenching, increases the relative proportion of carotenoids, and elevates antioxidant levels in mesophyll tissues, thereby establishing a multi-tiered protective system that includes light interception, thermal dissipation, and reactive oxygen species scavenging. This process, however, suppresses photosynthetic function, as evidenced by significant reductions in Pn, Gs, and ETR. Under LL conditions, the plant switches to a growth-first mode, leaves become broader, longer, and more horizontally oriented, chlorophyll content and the expression of photosynthesis-related genes are upregulated, and both light capture efficiency and carbon assimilation capacity are greatly enhanced (Figure 8). In this case, the plant prioritizes resource allocation to growth rather than defense. The spatial partitioning of leaf functions, with the adaxial side specializing in photosynthesis and the abaxial side specializing in photoprotection, effectively avoids resource competition between light energy utilization and stress defense, allowing *T. spathacea* to establish a dynamic balance between photoprotection and photosynthesis. This study not only clarifies the core mechanisms underlying the adaptation of this species to heterogeneous light environments but also provides a theoretical basis for understanding high-light adaptation strategies in shade-tolerant plants and for managing light environments in ornamental horticulture.

## Figures and Tables

**Table 1:** Primer design of internal and target genes.

Metabolic Pathway	Gene Name	Primer Sequence
Internal reference	*GAPDH*	Forward: 5′-CTGCTTCATTCAACATC-3′
		Reverse: 5′-CTCACGGTCAGATCAACA-3′
Anthocyanin	*PAL*	Forward: 5′-GTAGTCAACCTTGGCCTTGC-3′
		Reverse: 5′-TTTAGACCCCACCCCGTCAT-3′
	*CHS*	Forward: 5′-CGTACCCGTGCCCCTTTTAT-3′
		Reverse: 5′-CGGGTATGGTTCCCGTATCG-3′
	*CHI*	Forward: 5′-ACAGCTGGCTCAGTTGTCTC-3′
		Reverse: 5′-TCCCGTGGGGATGTCACTAT-3′
	*DFR*	Forward: 5′-CGGAGGAGGCTGGATTTCAC-3′
		Reverse: 5′-TCGATCTCTTTGTCCTCCGC-3′
Photosystem II	*PsbA*	Forward: 5′-TGGGAACTTAGCTTCTGCCTG-3′
		Reverse: 5′-GATCGAGAGGTTGTTCCGCC-3′
	*PsbO*	Forward: 5′-GCACTGGTTTGCTGTTCCAG-3′
		Reverse: 5′-CAGAGGGAGTGCCAAAGAGG-3′
	*PsbQ*	Forward: 5′-TTAGGAGCTGCTTCTGCTCAC-3′
		Reverse: 5′-ACTTGGGGACCTGTTTGGAC-3′
Photosystem I	*PsaD*	Forward: 5′-CCTTCCCTCATGATAGCCGC-3′
		Reverse: 5′-CGATCTTCGGAGGAAGCACC-3′
	*PsaF*	Forward: 5′-CAAGGCCTTCGACAAACGTG-3′
		Reverse: 5′-TAGTTGTCGAACCTGCGCTT-3′
	*PsaG*	Forward: 5′-AGCACAGGGCTCTCCCTATT-3′
		Reverse: 5′-TCCCTAGCCCTCACATCTCC-3′
Photosynthetic electron transport	*PetE*	Forward: 5′-GCCTCGTCTTTGTGCCTAGT-3′
		Reverse: 5′-ATTGACACCGGGTGGAACC-3′
	*PetF*	Forward: 5′-TTAGACGCTGCTGAGACTGC-3′
		Reverse: 5′-CCAGACACCATTTTGCCAGC-3′

## Data Availability

The original Illumina sequence and assembly sequence presented in the study are openly available in [NCBI’s Gene Expression Omnibus], and are accessible through GEO Series accession number GSE338282 at [https://www.ncbi.nlm.nih.gov/geo/query/acc.cgi?acc=GSE338282, accessed on 16 July 2026].

## Supplementary Materials

The following supporting information can be downloaded at https://www.mdpi.com/article/10.3390/ijms27146492/s1.
