# Supplementary material for: Survival Wisdom of Tradescantia spathacea Sw: Trade-Off Between Anthocyanin-Mediated Photoprotection and Photosynthetic Capacity Under High-Light Stress
Source: Int J Mol Sci. 2026 Jul 22;27(14):6492. doi: 10.3390/ijms27146492 (PMC13409763; doi:10.3390/ijms27146492)
Supplement: Supplementary file 1 [file ijms-27-06492-s001.zip › ijms-4414814-supplementary.pdf]

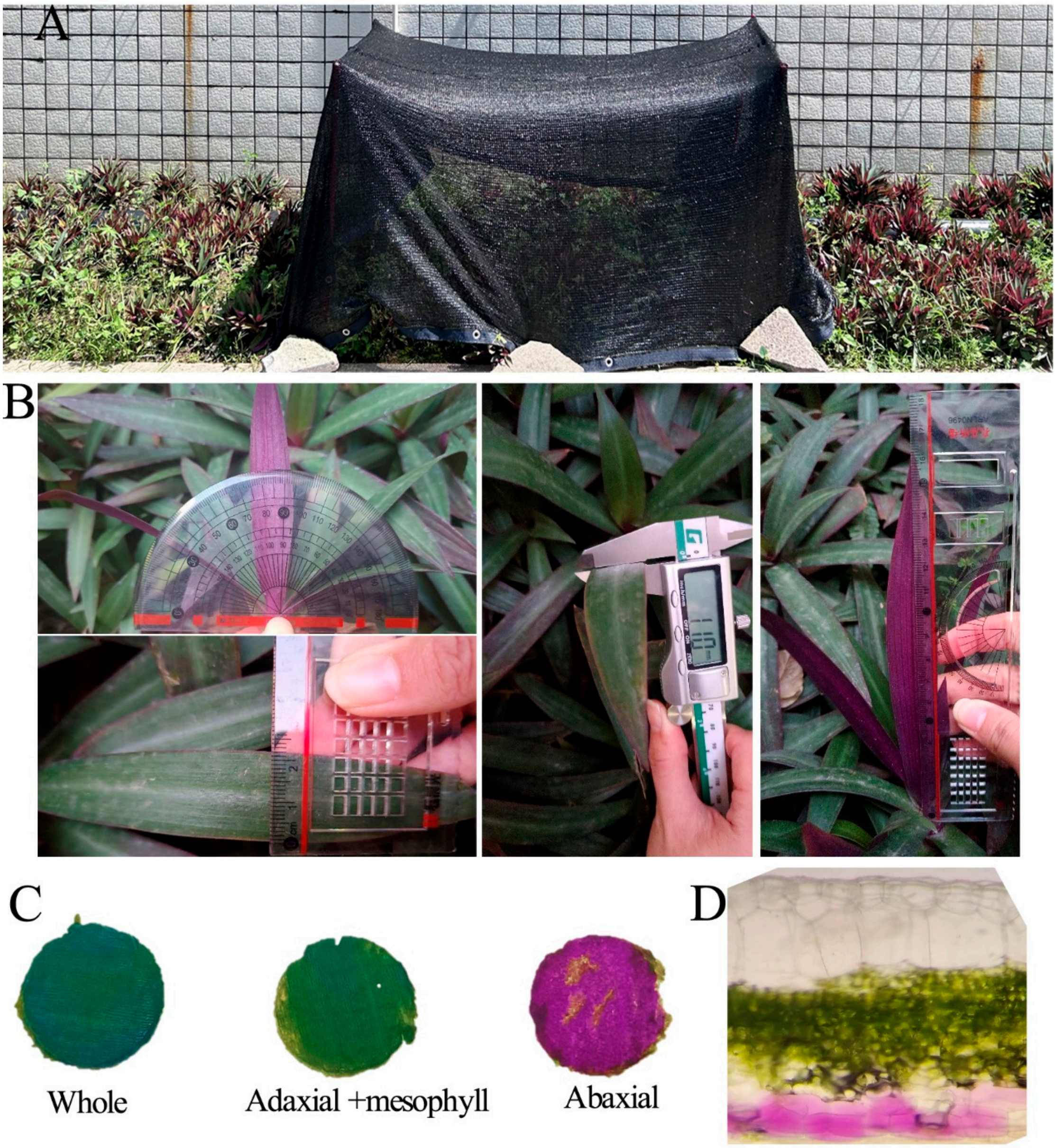

Figure S1. Some photos of plants during the planting, processing and measurement processes. (A) The treatment diagram of *T.spathacea* under FL and LL conditions. (B) The process diagram of measuring phenotypic indicators of *T.spathacea*. (C) Photos of leaf sampling and tissue separation (adaxial epidermis + mesophyll cells, abaxial epidermis). (D) The adaxial epidermis and abaxial epidermis are fully displayed to show the cross-sectional view of the leaf.

Table S1. Sample Sequencing quality control results of *T. spathacea* under different light conditions

Samples	Read Number	Base Number	GC Content	%≥Q30
FL1	19,408,233	5,801,712,497	41.30%	98.22%
FL2	20,803,209	6,221,534,621	41.71%	97.76%
FL3	19,589,543	5,855,105,941	41.51%	97.91%
LL1	19,454,865	5,818,110,827	42.45%	97.88%

LL2	19,582,579	5,859,775,809	42.02%	97.85%
LL3	19,396,141	5,805,166,660	42.19%	98.10%

Notes: Read Number represents the total number of pair end Reads in Clean Data; Base Number represents the total base number of Clean Data; GC Content represents the percentage of total bases in Clean Data that are G and C bases; The %≥Q30 represents the percentage of bases with Clean Data quality values greater than or equal to 30

Table S2. Statistical table of assembly results *T. spathacea* under different light conditions

Length Range	Transcript	Unigene
200-300	18,850(8.50%)	12,562(13.66%)
300-500	44,027(19.86%)	23,968(26.06%)
500-1000	61,298(27.65%)	25,000(27.18%)
1000-2000	55,558(25.06%)	16,595(18.04%)
2000+	41,995(18.94%)	13,862(15.07%)
Total Number	221,728	91,987
Total Length	277,058,432	98,518,706
N50 Length	1,903	1,790
Mean Length	1249.54	1071.01

Notes: Length Range represents the different length intervals of the Unigene. The numbers in the table indicate the number of Unigenes in the corresponding intervals, and the percentages in parentheses indicate the proportion of Unigenes in the corresponding length intervals. Total Number denotes the total number of Unigenes assembled; Total Length denotes the total length of Unigenes assembled; N50Length denotes the length of N50 of Unigenes; Mean Length denotes the average length of Unigenes

Table S3. Comparison statistics of sequencing data and assembly results of *T. spathacea* under different light conditions

Samples	Clean Reads	Mapped Reads	Mapped Ratio
FL1	19,408,233	14,749,963	76.00%
FL2	20,803,209	15,960,405	76.72%
FL3	19,589,543	14,963,120	76.38%
LL1	19,454,865	15,085,839	77.54%

LL2	19,582,579	14,962,862	76.41%
LL3	19,396,141	14,973,257	77.20%

Notes: Clean Reads denotes the number of Clean Reads in double-ended; Mapped Reads denotes the number of Mapped Reads in double-ended; and Mapped Ratio denotes the proportion of Mapped Reads in Clean Reads

Table S4. Unigene annotation statistics table of *T. spathacea* under different light conditions

Anno_Database	Annotated_Number	300<=length<1000	length>=1000
COG_Annotation	7754	1788	5718
GO_Annotation	32294	11441	18800
KEGG_Annotation	24832	8132	15184
KOG_Annotation	20221	6471	12555
Pfam_Annotation	22084	6434	14804
Swissprot_Annotation	25103	8148	15536
TrEMBL_Annotation	37969	13790	21768
eggNOG_Annotation	32589	11456	19213
nr_Annotation	37708	13666	21690
All_Annotated	39546	14677	22260

Notes: Anno databases denotes the respective functional databases; Annotated Number denotes the number of Unigene annotated to the database; 300≤length indicates the number of assembled Unigenes whose length is less than or equal to 300; length≥1000 indicates the number of assembled Unigenes with length greater than or equal to 1000
